# Optimisation of the Monitoring Strategy of Macroinvertebrate Communities in the River Dender, in Relation to the EU Water Framework Directive

**DOI:** 10.1100/tsw.2002.121

**Published:** 2002-03-08

**Authors:** Tom P. D’heygere, Peter L.M. Goethals, Niels De Pauw

**Affiliations:** Laboratory of Environmental Toxicology and Aquatic Ecology, Ghent University, J. Plateaustraat 22, B-9000 Gent, Belgium

**Keywords:** Water Framework Directive, macroinvertebrates, ecological modelling classification trees

## Abstract

The Dender basin in Flanders (Belgium) was used as a case study to implement the European Union (EU) Water Framework Directive. During the last 5 years, ample research on pollution loads and ecological water quality has been done on the Dender River. In addition to biological sampling of macroinvertebrates and fish, automated measurement stations were also used to investigate the spatial-temporal variability of the physical-chemical water quality. This research revealed that the pollution of the Dender River is highly variable. The high nutrient loads result in severe algae blooms during summer, leading to very complex diurnal processes. In this paper, the monitoring strategy for the assessment of the biological water quality in the Dender basin has been reviewed in relation to the EU Water Framework Directive. For this, seasonal macroinvertebrate data were collected and assessed. General trends and hidden structures in these data were analysed by means of classification trees, using different inputs (seasons, river types, and subbasins). Validation of the results was obtained by applying statistical methods. Analysis about the presence and abundance of the macroinvertebrates revealed that there is a distinct difference between the biological water quality in the Dender stem river and its tributaries. There are also seasonal differences between the macroinvertebrate communities when the Dender and its tributaries are examined separately. An optimised monitoring strategy is proposed based on these results and the EU Water Framework Directive. This includes two monitoring campaigns in summer and winter every 3 years. Furthermore, a cyclic monitoring scheme was developed to minimise sampling efforts.

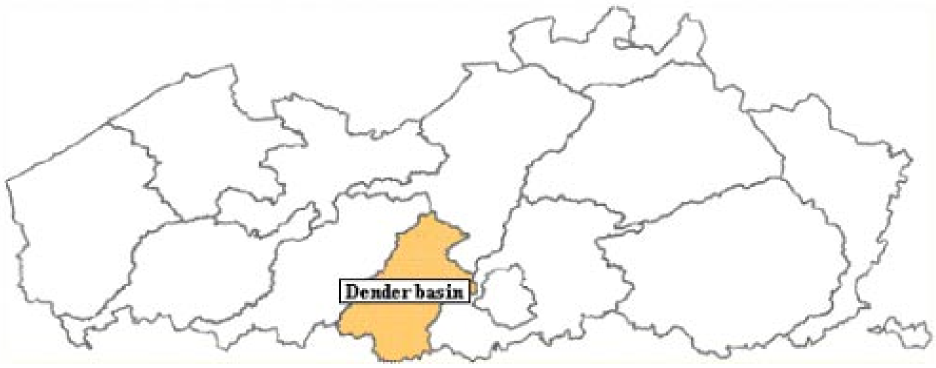

**FIGURE 1.** The Dender River basin in Flanders.

## INTRODUCTION

Monitoring is one of the crucial factors in assessing river systems. The management quality largely depends upon the efficacy and efficiency of the monitoring process. Defining the information need is the basis for developing an optimal monitoring strategy[1]. Therefore, the European Union (EU) has adopted the Water Framework Directive[2]. This Directive provides several guidelines for monitoring our water systems, but local governments still have considerable freedom regarding the practical implementation of the Directive. Each European country, therefore, must develop a consistent monitoring strategy and assessment methodology[3].

The Dender basin (708 km²) in Flanders (Belgium) is used to implement the Directive (Fig. 1)[4]. More than 90% of the surface water of the Dender consists of rainwater, causing an irregular flow. To allow boat traffic and prevent floods, several weirs were constructed on the Dender stem river and large parts were canalized. During summer, the flow is consequently reduced to nearly zero, allowing large algae blooms to develop[5]. Agriculture, industrial, and domestic wastewater have a large influence on the pollution load and eutrophic state of the water system. The data of seasonal monitoring campaigns of the macroinvertebrate communities will be used in this paper to develop an optimised monitoring strategy in the Dender basin.

## EXPERIMENTAL METHODS AND PROCEDURES

The assessment of the water quality was based on the Belgian Biotic Index (BBI). This biological assessment method uses macroinvertebrates as indicators for the pollution level and is a standardised method in Belgium[6]. The scoring system ranges from 10 to 0. It is combined with a colour code: 9–10 = unpolluted (blue), 7–8 = slightly polluted (green), 5–6 = moderately polluted (yellow), 3–4 = heavily polluted (orange), 1–2 = very heavily polluted (red), 0 = dead (black). The score system is based on the theorem that increasing pollution will result in a loss of diversity and a progressive elimination of certain pollution-sensitive groups[7,8]. The macroinvertebrate communities were sampled by means of *in situ* exposure of artificial substrates instead of a handnet, which is commonly used in Flanders when possible. For deeper rivers, such as the Dender, it was shown that the use of artificial substrates can provide a valid alternative to handnet sampling of the macroinvertebrate sampling[9,10]. The macroinvertebrates were identified at the level required for the assessment of the water quality by the BBI and counted[6].

A total of 22 measuring sites were selected on a river stretch in the Dender basin of about 25 km between Geraardsbergen and Denderleeuw: 14 in the Dender stem river and 8 on the most important tributaries (Fig. 2). These sites were sampled during five monitoring campaigns in

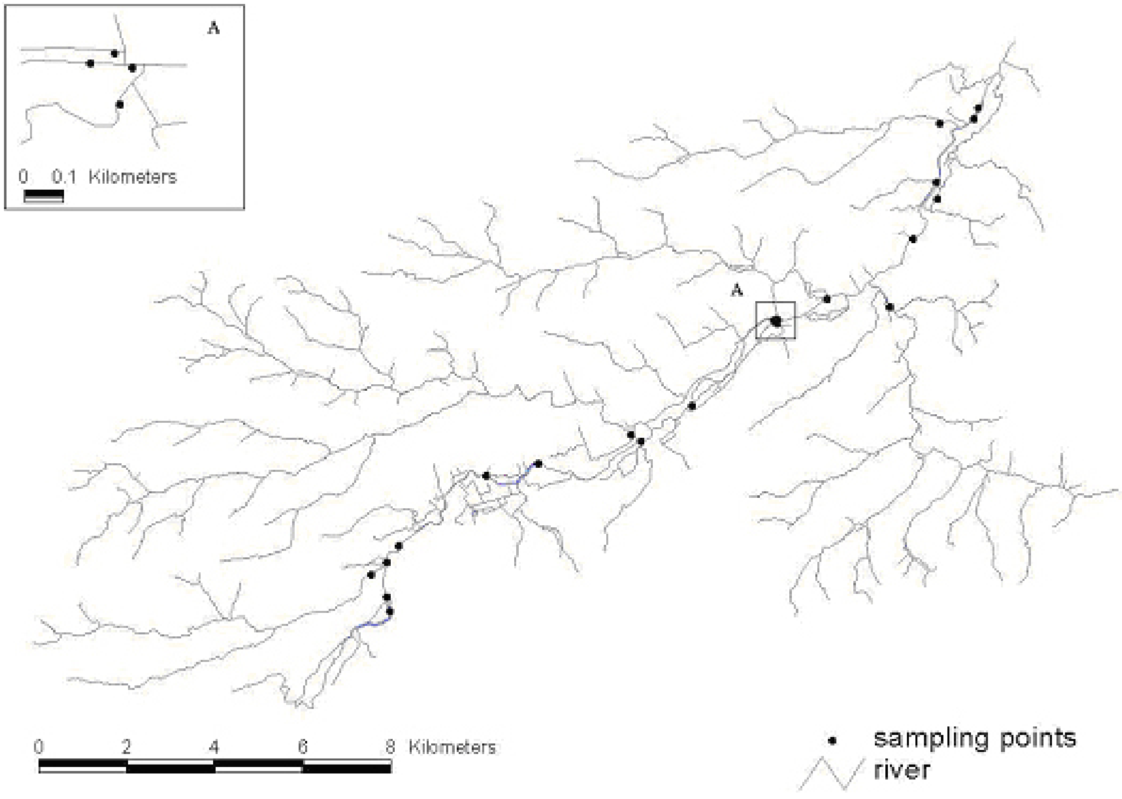

**FIGURE 2.** Selected sampling sites in the Dender River basin.

consecutive seasons, from the summer of 1999 till the summer of 2000. The first monitoring campaign took place in the months August–September 1999 (summer) and the following campaigns in October–November (autumn), January–February (winter), March–April (spring), and July–August (summer).

The collected data were analysed by classification trees, a machine learning technique[11]. This method generates results with a low prediction error and allows the user to identify associations and general trends in the data, making it more interesting than complete black-box techniques such as artificial networks[12]. In this study, Classification and Regression Trees (CART) was used to induce classification trees and is technically known as binary recursive partitioning methodology. The process is binary because parent nodes are always separated into exactly two child nodes by splitting rules, based on a question relating to a single variable. This methodology is recursive because the process can be repeated by treating each child node as a parent. CART proceeds by growing trees until a maximal tree is generated and a class outcome is assigned to each terminal node. Postpruning prevents trees from overfitting the data[11,13].

The input variables were "river type" (Dender stem river or its tributaries), "season" (summer, autumn, winter, and spring), and "basin" (four basins on the river stretch, divided by flow control weirs). The output variables, the collected macroinvertebrates, were discrete: absent, present (≤ 100), or abundantly present (> 100).

The structures in the data, revealed by using classification trees, were tested statistically using the BBI values as input values instead of the macroinvertebrates. Statistical Products and Service Solutions (SPSS) was used for the analysis. It is a reliable tool for data processing, which can quickly analyse large datasets. An "Independent-Samples T-test" compares the average BBI value of the primary splits in the classification trees, taking the standard deviation into account. A

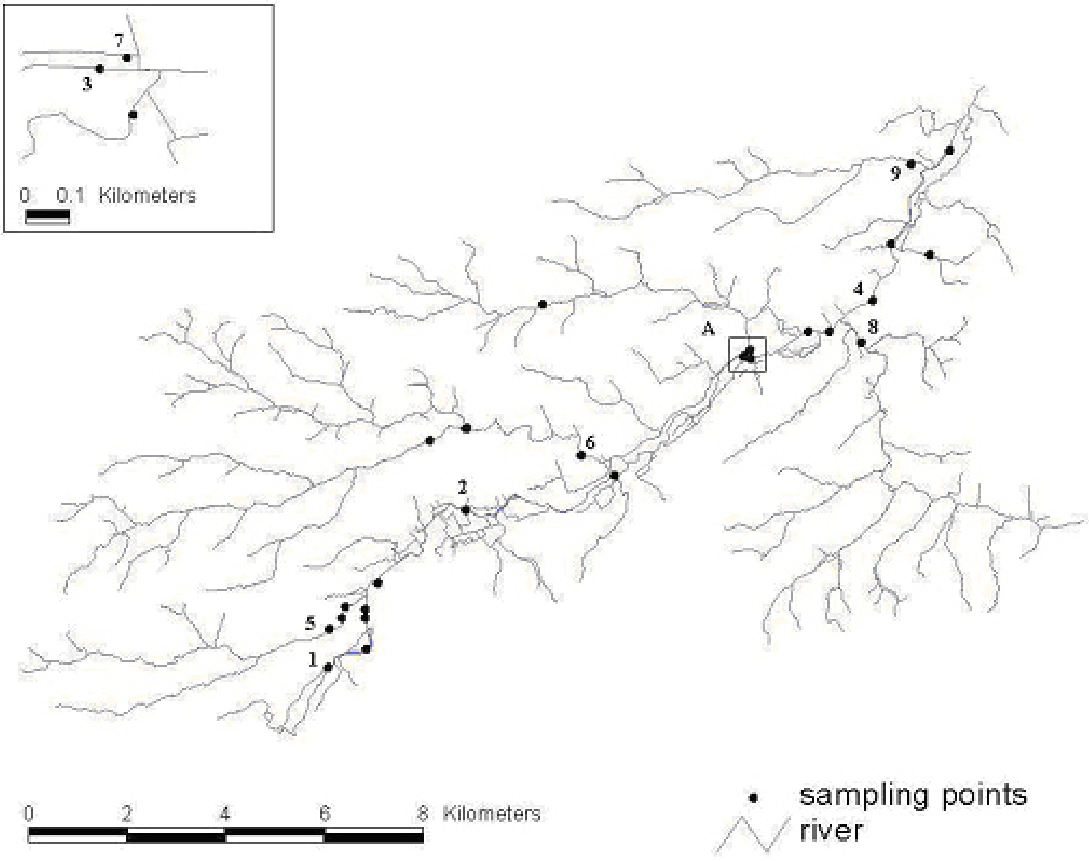

**FIGURE 3.** Sampling sites of the Flemish Environmental Agency (VMM) in the Dender basin.

visual presentation of the results is given by boxplots: the horizontal line in the box represents the median value while the boundary of the boxplot shows the 25th and 75th quartile. The whiskers represent the lowest values that are not outliers[14].

## RESULTS AND DISCUSSION

### Evolution of the Biological Water Quality

The evolution of the biological water quality during the period 1992–2000 was assessed by the BBI data of the Flemish Environmental Agency (VMM) in the Dender basin. The sampling sites of the VMM in the Dender River basin are represented in Fig. 3, and the evolution of the yearly BBI measurements (1992–1999) in a selection of sampling sites is shown in Fig. 4. The numbers 1 to 9 in Fig. 4 correspond with the numbered sampling sites in Fig. 3. The BBI was almost constant in several sampling sites while in other sites the BBI strongly fluctuated, but no significant change in water quality could be detected.

In the Water Framework Directive, it is stated that an assessment of the ecological water quality is to be repeated at 3-year intervals for surveillance monitoring, macroinvertebrates included[2]. When the ecological water quality evolves very slowly or not at all, a lower monitoring frequency is advisable. The evolution of the BBI for yearly and 3-yearly monitoring (by removing measurements) was therefore compared in Fig. 5. In both cases, a normal distribution

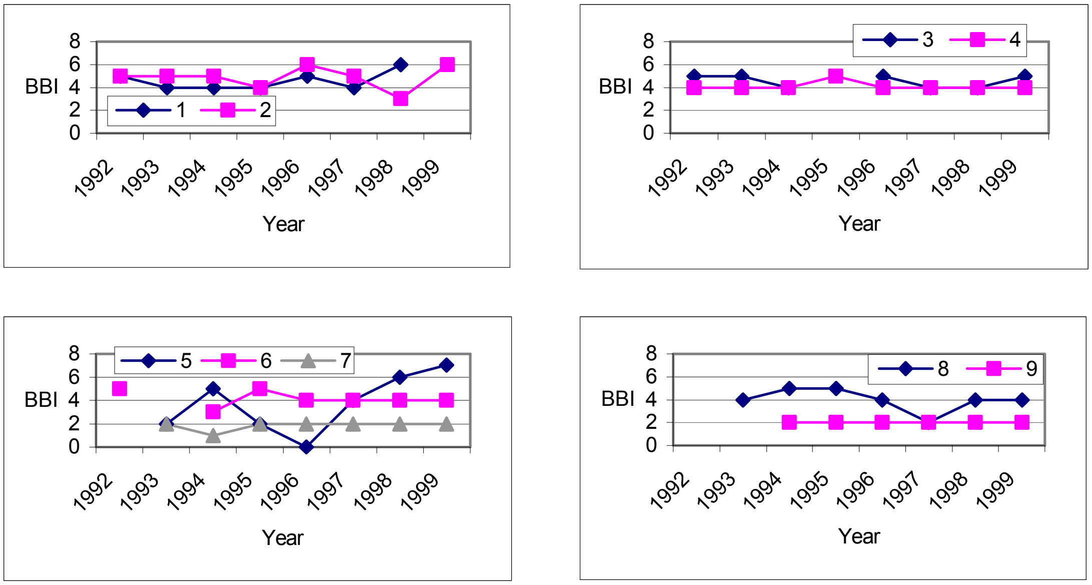

**FIGURE 4.** Evolution of the BBI in a selection of sampling sites of the VMM in the Dender basin (1 to 9).

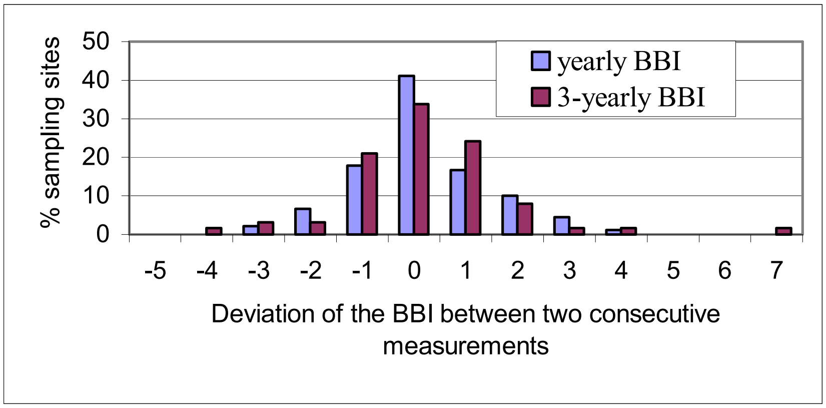

**FIGURE 5.** Influence of the monitoring frequency on the deviation between two consecutive BBI measurements.

could be observed with maxima at zero deviation between two consecutive measurements. A large deviation in the BBI (3 to 4) was in most cases annulated the following year. In 76 and 79% of the cases for respectively yearly and 3-yearly monitoring, a maximum deviation of one BBI unit was observed. This indicated that an update of the biological assessment every 3 years is sufficient.

## Modelling

The data of the macroinvertebrates were divided into three classes (absent, present, and abundantly present) and used to produce classification trees with the input variables (river type and season). A classification tree could not be produced for every taxon because some taxa were only found once or twice, e.g., Mollusca such as *Ancylus, Gyraulus, Sphaerium,* and *Planorbarius.* Therefore, the different Mollusca taxa were considered together. This was also the case for the Ephemeroptera and Hirudinea taxa. For the same reason, trees could only be constructed for the Diptera species *Chironomus thummi-plumosus* and *C. non thummi-plumosus.*

The results revealed a clear distinction between taxa in the Dender stem river and the tributaries. A second split divided the taxa into different seasons, but it seemed that the second split was particularly related to the difference between summer and winter. Removing the data gathered in autumn and spring improved the prediction success. Fig. 6 represents a classification tree for Mollusca. Mollusca were found in eight sampling sites between summer and winter, but never abundantly (no class 2). A first split showed that if Mollusca were present, this mainly was in the tributaries (33.3 ↔ 5.4%), and in the tributaries Mollusca were found in the summer (45.4 ↔ 14.3%). In two out of the three cases where Mollusca were present and classified as not present, it concerned *Sphaerium*, which is less sensitive for pollution than other Mollusca.

The results for all macroinvertebrate taxa are shown in Table 1. In every classification tree, a split occurred between the Dender stem river and its tributaries. No seasonal split could be found for Tubificidae, Gammaridae, and *Ischnura.* In the Dender stem river, Hirudinea species occurred abundantly in the summer (75, 100, and 84.2% of all cases classified as class 2 for *Erpobdella, Glossiphonia,* and *Helobdella,* respectively). When the variable "basin" was applied, classification trees revealed a difference in basin 1&4 and basin 2&3 for Lumbricidae, Gammaridae, *Chironomus thummi-plumosus,* and for the Hirudinea genera *Glossiphonia, Helobdella, Hemiclepsis,* and *Piscicola*.

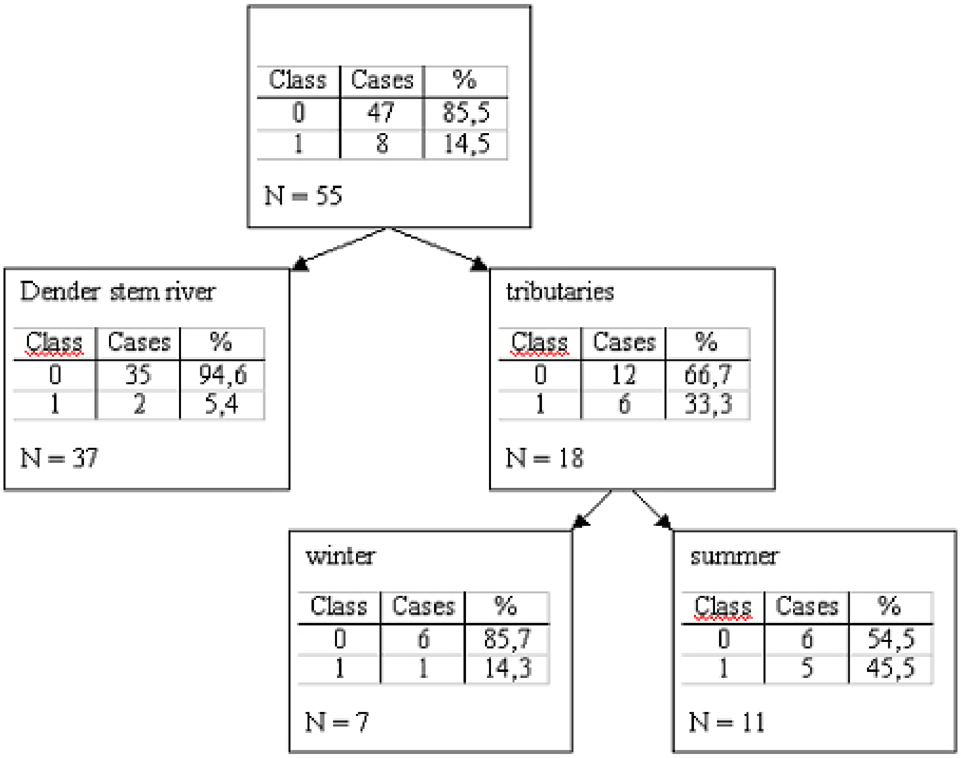

**FIGURE 6.** Classification tree for Mollusca with splitting variables "river type" (Dender stem river) and "season" (only summer and winter). "N" represents the total number of measuring cases that are present in a branch of the classification tree, divided over "class 0" (no Mollusca present) and "class 1". (Mollusca present, but not abundantly [< 100]). No class 2 cases (Mollusca abundantly present) were found.

**TABLE 1**
**Classification of Macroinvertebrate Taxa According to River Type and Season (Class 0 = Absent; Class 1 = Present; Class 2 = Abundantly Present)**

| | Dender Stem River | | Tributary | |
|---|---|---|---|---|
| | **Summer** | **Winter** | **Summer** | **Winter** |
| Tubificidae | No classification tree | | | |
| Lumbricidae | Class 0: 94.6% | | Class 0: 90.9% | Class 1: 57.1% |
| | Class 2: 87% | Class 1 and 2: 100% | Class 0 and 1: 100% | |
| **HIRUDINEA** | | | | |
| *Erpobdella* | Class 1 and 2: 100% | Class 1: 78.6% | Class 0: 72.2% | |
| *Glossiphonia* | Class 1 and 2: 83% | Class 1: 92.9% | Class 0: 61.1% | |
| *Helobdella* | Class 1 and 2: 96% | Class 1: 71.4% | Class 0: 66.7% | |
| *Hemiclepsis* | Class 1: 69.6% | Class 0: 85.7% | Class 0: 94.4% | |
| *Piscicola* | Class 1: 13,0% | Class 0: 100% | Class 0: 100% | |
| Gammaridae | Class 1: 8.7% | Class 0: 100% | Class 0: 100% | |
| Asellidae | Class 2: 83.8% | | Class 0 and 1: 94.4% | |
| *Ischnura* | Class 1: 45.9% | | Class 0: 100% | |
| | Class 0: 94.6% | | Class 1: 54.5% | Class 0: 85.7% |
| **MOLLUSCA** | | | | |
| *Lymnaea* | Class 0 : 100% | | Class 1 : 27,3% | Class 0: 100% |
| *Physa* | Class 0 : 97.3% | | Class 1 : 27.3% | Class 0: 100% |
| *Armiger* | Class 0 : 100% | | Class 1 : 27.3% | Class 0: 100% |
| | Class 0 : 100% | | Class 1 : 18.2% | Class 0: 100% |
| **Coleoptera** | | | | |
| | Class 1 : 13% | Class 0: 100% | Class 0 : 100% | |
| **EPHEMEROPTERA** | | | | |
| *Chironomus thummi-plumosus* | Class 0 : 77.8% | Class 2 : 64.3% | Class 2 : 66.7% | |
| *Chironomus non thummi-plumosus* | Class 2 : 91.9% | | Class 0 and 1: 81.9% | Class 1: 83.3% |

## Statistical Approach

It is important that the shifts in the macroinvertebrate communities, revealed by means of classification trees, are also translated into the BBI. This will indicate the importance of the general trends in the data. By means of an Independent-Samples T-test, a significant difference between the BBI in the Dender stem river and its tributaries (variable "river type") was found, also when the VMM data between 1997 and 1999 were used. A significant difference between summer and winter could only be detected when making a distinction between the Dender River and its tributaries. Then a significant difference between summer and winter was found in the tributaries, but not in the Dender stem river (Fig. 7). This is probably caused by the variance of the BBI method itself. The most sensitive macroinvertebrates for pollution in the tributaries were the Mollusca, which were mainly found in the summer. The BBI in the winter will therefore be lower. In the Dender stem river, more macroinvertebrate taxa with the same sensitivity were present. The BBI is consequently more stable because different taxa lead to the same BBI value. There was also a significant difference observed between the BBI of basin 1&4 and basin 2&3. The data of all seasons were used because there was no significant difference between the seasons in the Dender stem river itself. When using the VMM data between 1997 and 1999, this difference was not observed.

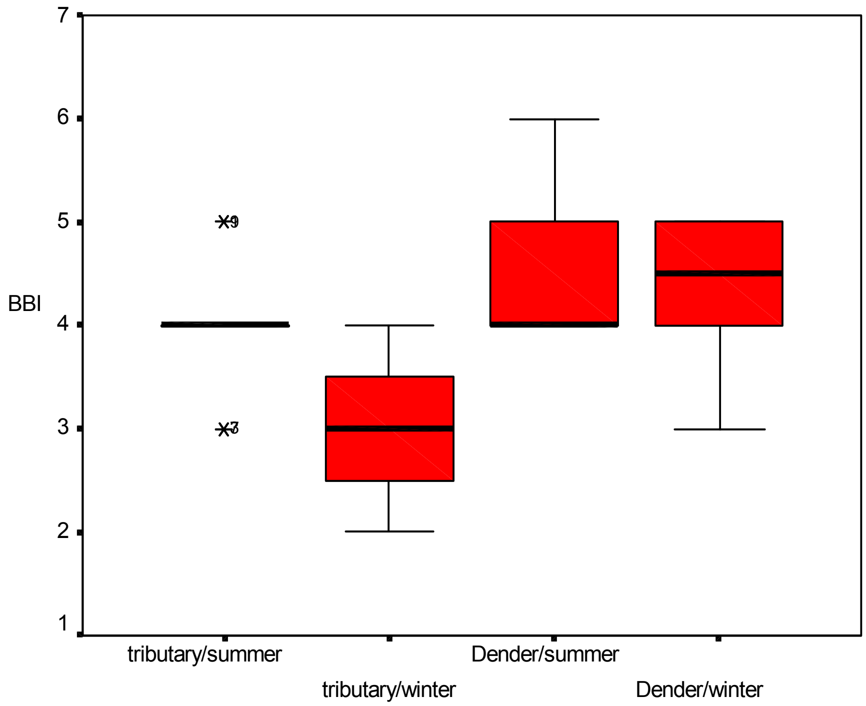

**FIGURE 7.** Boxplot of the BBI in summer and winter after splitting according to "river type".

## Water Framework Directive

In the Water Framework Directive, 3-year intervals for surveillance monitoring were suggested for the assessment of ecological water quality. An assessment of the yearly BBI measurements in the Dender basin during the 1990s indicated that a monitoring frequency of 3 years for macroinvertebrates is high enough. The Directive states that for any parameter a suited monitoring frequency is required to provide data for a reliable assessment of the status of the quality element[2]. On the basis of the BBI, a lower water quality was discovered in winter, especially in the tributaries. To get a reliable image of the biological water quality, biological monitoring should be carried out twice a year. Due to the annual dynamics of streams, the U.S. Environmental Protection Agency states that sampling should be carried out at various seasons to include the major components of the spring-summer and autumn-winter communities[15,16]. Biological sampling twice annually is also applied in the Transnational Monitoring Network (TNMN) in the Danube River basin[17].

The Directive furthermore stated that the times at which monitoring is undertaken should be selected to minimise the impact of seasonal variation on the results, thus ensuring that the results reflect changes in the water body due to changes in antropogenic pressure. Additional monitoring during different seasons of the same year should be carried out, when necessary, to achieve this objective[2]. To what extent shifts in the macroinvertebrate communities during summer and winter are due to seasonal influences or antropogenic pressure is not yet known. Overall it is suggested, based on the results and according to the Directive, to assess the ecological water quality at 3-year intervals for surveillance monitoring, but sampling twice a year when surveillance monitoring is carried out.

The modified monitoring frequency for surveillance monitoring, compared with the VMM, does not necessarily affect the sampling effort (personnel and resources). With the same density of sampling sites, the sampling effort is lower if monitoring is carried out twice annually for every 3 years instead of once annually for every year. Operational and investigative monitoring

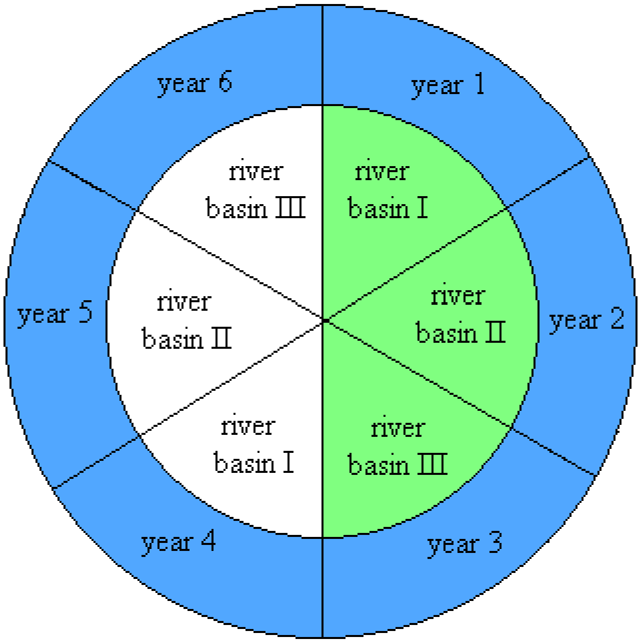

**FIGURE 8.** Monitoring scheme with three river basins. (Green shaded area = river basin management plan required.)

are not considered. By introducing the monitoring scheme presented in Fig. 8, the sampling effort is evenly spread in time. Per three river basins, one river basin is sampled every year. Consequently, the time frame in which all samples can be taken is much smaller when only one third of the total sampling sites have to be sampled (one out of three river basins). Not only is the sampling effort lower, but also the seasonal variability can be reduced. The monitoring scheme also takes into account that river basin management plans have to be produced every 6 years.

Nowadays, an increasing attention is paid to ecological modelling. Models might be interesting to reduce subjectivity of score systems, to allow a better interpretation of the results, to come to a cause-allocation of the state of a river, and to increase the insight necessary to improve assessment systems[3]. Additional monitoring in winter could provide valuable input for ecological models, regarding the presence and shifts in the abundance of macroinvertebrates. Further studies about the application of other biological quality elements regarding the Water Framework Directive still have to be conducted. The Fish Index or Index of Biotic Integrity (IBI)[18] is still under development for Flemish watercourses. Recently research is being done on assessment methods based on diatoms and macrophytes[19]. These biological quality elements will also be assessed on the basis of an index, combined with a colour code.

## CONCLUSIONS

Seasonal macroinvertebrate data of a river stretch in the Dender basin were analysed by means of classification trees and validated statistically. A distinction between the ecological water quality of the Dender stem river and its tributaries was found. To obtain a reliable assessment of the ecological water quality, it was shown that monitoring campaigns should be carried out twice annually (summer and winter) to cope with the seasonal variability. Implementation of the Water Framework Directive led to a cyclic monitoring scheme with a 3-year period to minimise the sampling efforts.

## ACKNOWLEDGEMENTS

The first author is a recipient of a grant of the Institute for the Promotion of Innovation by Science and Technology in Flanders (IWT).
